# Genome-Wide Interaction with Insulin Secretion Loci Reveals Novel Loci for Type 2 Diabetes in African Americans

**DOI:** 10.1371/journal.pone.0159977

**Published:** 2016-07-22

**Authors:** Jacob M. Keaton, Jacklyn N. Hellwege, Maggie C. Y. Ng, Nicholette D. Palmer, James S. Pankow, Myriam Fornage, James G. Wilson, Adolfo Correa, Laura J. Rasmussen-Torvik, Jerome I. Rotter, Yii-Der I. Chen, Kent D. Taylor, Stephen S. Rich, Lynne E. Wagenknecht, Barry I. Freedman, Donald W. Bowden

**Affiliations:** 1 Molecular Genetics and Genomics Program, Wake Forest School of Medicine, Winston-Salem, North Carolina, United States of America; 2 Center for Genomics and Personalized Medicine Research, Wake Forest School of Medicine, Winston-Salem, North Carolina, United States of America; 3 Center for Diabetes Research, Wake Forest School of Medicine, Winston-Salem, North Carolina, United States of America; 4 Department of Biochemistry, Wake Forest School of Medicine, Winston-Salem, North Carolina, United States of America; 5 Center for Public Health Genomics, Wake Forest School of Medicine, Winston-Salem, North Carolina, United States of America; 6 Division of Epidemiology and Community Health, University of Minnesota, Minneapolis, Minnesota, United States of America; 7 Institute of Molecular Medicine and Human Genetics Center, University of Texas Health Science Center at Houston, Houston, Texas, United States of America; 8 University of Mississippi Medical Center, Jackson, Mississippi, United States of America; 9 Department of Preventive Medicine, Northwestern University Feinberg School of Medicine, Chicago, Illinois, United States of America; 10 Institute for Translational Genomics and Population Sciences, Los Angeles BioMedical Research Institute, Harbor-UCLA Medical Center, Torrance, California, United States of America; 11 Center for Public Health Genomics, University of Virginia, Charlottesville, Virginia, United States of America; 12 Division of Public Health Sciences, Wake Forest School of Medicine, Winston-Salem, North Carolina, United States of America; 13 Department of Internal Medicine - Section on Nephrology, Wake Forest School of Medicine, Winston-Salem, North Carolina, United States of America; The University of Texas School of Public Health, UNITED STATES

## Abstract

Type 2 diabetes (T2D) is the result of metabolic defects in insulin secretion and insulin sensitivity, yet most T2D loci identified to date influence insulin secretion. We hypothesized that T2D loci, particularly those affecting insulin sensitivity, can be identified through interaction with insulin secretion loci. To test this hypothesis, single nucleotide polymorphisms (SNPs) associated with acute insulin response to glucose (AIR_g_), a dynamic measure of first-phase insulin secretion, were identified in African Americans from the Insulin Resistance Atherosclerosis Family Study (IRASFS; n = 492 subjects). These SNPs were tested for interaction, individually and jointly as a genetic risk score (GRS), using genome-wide association study (GWAS) data from five cohorts (ARIC, CARDIA, JHS, MESA, WFSM; n = 2,725 cases, 4,167 controls) with T2D as the outcome. In single variant analyses, suggestively significant (*P*_interaction_<5×10^−6^) interactions were observed at several loci including *LYPLAL1* (rs10746381), *CHN2* (rs7796525), and *EXOC1* (rs4289500). Notable AIR_g_ GRS interactions were observed with *SAMD4A* (rs11627203) and *UTRN* (rs17074194). These data support the hypothesis that additional genetic factors contributing to T2D risk can be identified by interactions with insulin secretion loci.

## Introduction

Although common variants examined in genome-wide association studies (GWAS) have identified ~80 loci associated with T2D risk, these variants explain only about 15% of T2D heritability [1,2]. A portion of the missing heritability may be explained by epistasis, which occurs when a genetic risk factor is modified by other factors in an individual’s genetic background [3]. Epistasis, or gene-gene interaction, analyses may facilitate the detection of novel loci when non-additive effects exist, but may also provide novel insights illuminating biological mechanisms underlying complex diseases such as T2D [4].

T2D is characterized by impaired insulin secretion arising from pancreatic beta-cell dysfunction and insulin resistance in hepatic, skeletal muscle, and other peripheral tissues, leading to decreased plasma glucose uptake. However, documented T2D loci primarily map to genes influencing insulin secretion or other aspects of beta-cell biology [1]. Given the underlying bimodal pathophysiology, T2D may be a particularly well-suited disease model for hypothesis-driven investigation of epistatic interactions. Genetic insults to both insulin secretion and insulin sensitivity may jointly increase an individual’s T2D risk in a non-additive manner. Considering the higher prevalence rate of T2D, insulin resistance, and obesity, African Americans are optimal for the study of genetic interactions that contribute to T2D risk.

In an effort to identify interactions contributing to T2D and to discover novel insulin sensitivity loci, we hypothesized that T2D risk loci, particularly those affecting insulin sensitivity, could be identified by interaction analyses with insulin secretion loci. In cross-sectional meta-analyses of five T2D studies (ARIC, CARDIA, JHS, MESA, and WFSM), we tested whether 5 insulin secretion SNPs, or a genetic risk score summarizing these SNPs, modified genome-wide SNP associations with T2D risk.

## Research Design and Methods

### Subjects

Two sources of data were analyzed in this study. Primary inferences of association with insulin secretion were derived from African American participants (n = 492 individuals from 42 families) in the Insulin Resistance Atherosclerosis Family Study (IRASFS), a metabolically well-characterized cohort [5]. Glucose homeostasis traits were measured by the frequently sampled intravenous glucose tolerance test (FSIGT) [5]. Briefly, a 50% glucose solution (0.3g/kg) and regular human insulin (0.03units/kg) were injected intravenously at 0 and 20 minutes, respectively. Blood was collected at −5, 2, 4, 8, 19, 22, 30, 40, 50, 70, 100, and 180 minutes for measurement of plasma glucose and insulin. AIR_g_ was calculated as the increase in insulin at 2–8 minutes above the basal (fasting) insulin level after the bolus glucose injection at 0–1 minute. Insulin sensitivity (S_I_) was calculated by mathematical modeling using the MINMOD program (version 3.0 [1994]) [6]. Disposition index (DI) was calculated as the product of S_I_ and AIR_g_.

Inferences of genome-wide epistatic interaction with insulin secretion loci for T2D susceptibility were derived from African American participants from the Atherosclerosis Risk in Communities Study (ARIC; n = 955 T2D cases, 414 controls), Coronary Artery Risk Development in Young Adults (CARDIA; n = 94 T2D cases, 654 controls), Jackson Heart Study (JHS; n = 333 T2D cases, 1,450 controls), Multi-Ethnic Study of Atherosclerosis (MESA; n = 411 T2D cases, 793 controls), and the Wake Forest School of Medicine (WFSM; n = 932 T2D cases, 856 controls) cohorts for a total of 2,725 T2D cases and 4,167 controls [7–12]. T2D was diagnosed according to the American Diabetes Association criteria with at least one of the following: fasting glucose ≥126 mg/dL, 2-h oral glucose tolerance test glucose ≥200 mg/dL, random glucose ≥200 mg/dL, use of oral hypoglycemic agents and/or insulin, or physician diagnosed diabetes. Subjects diagnosed before 25 years of age were excluded. Normal glucose tolerance was defined as fasting glucose <100 mg/dL and 2-h oral glucose tolerance test glucose <140 mg/dL (if available) without reported use of diabetes medications. Control subjects <25 years of age were excluded.

IRB approval was obtained at all sites and all participants provided written informed consent. Descriptions of the T2D study cohorts and funding sources for each individual study are summarized in S1 Appendix.

### Genotyping, imputation, and quality control

For the IRASFS samples, genotyping and quality control were performed at the Wake Forest Center for Genomics and Personalized Medicine Research using the Illumina Infinium HumanExome BeadChip v1.0 as previously described [13]. Briefly, the exome chip contained 247,870 variants (92% protein coding), In addition, the chip included 64 SNPs associated with T2D from previous GWAS in Europeans, many of which have been implicated in insulin secretion (exome chip design: http://genome.sph.umich.edu/wiki/Exome_Chip_Design). Sample and autosomal SNP call rates were ≥99%, and SNPs with poor cluster separation (<0.35) were excluded. Mendelian errors were identified using PedCheck [14] and resolved by removing conflicting genotypes. Hardy–Weinberg Equilibrium (HWE) was assessed in unrelated samples (n = 39) using PLINK (http://pngu.mgh.harvard.edu/purcell/plink) [15] to reduce biases introduced by familial allele frequencies. All variants were in accordance with HWE (*P*>1x10^-5^).

As described in detail previously[16], the T2D study samples were genotyped using the Affymetrix Genome-Wide Human SNP Array 6.0. For the ARIC, CARDIA, JHS, and MESA cohorts, genotyping and quality control were completed by the National Heart, Lung, and Blood Institute’s (NHLBI’s) Candidate Gene Association Resource (CARe) at the Broad Institute [17]. Genotyping for the WFSM study was performed at the Center for Inherited Disease Research (CIDR). For all T2D studies, imputation was performed using MACH with the function–mle (version 1.0.16, http://www.sph.umich.edu/csg/abecasis/MaCH/) to obtain missing genotypes and replace genotypes inconsistent with reference haplotypes as previously described [18]. SNPs with call rate ≥95% and minor allele frequency (MAF) ≥1% that passed study-specific quality control were used for imputation [17,19]. A 1:1 HapMap II (NCBI Build 36) CEU:YRI (European:African) consensus haplotype was used as reference. A total of 2,713,329 to 2,907,086 autosomal SNPs from each GWAS with call rate ≥95%, MAF ≥1%, and Hardy-Weinberg P-value ≥0.0001 for genotyped SNPs and MAF ≥1% and RSQ ≥0.5 for imputed SNPs were included in subsequent data analyses.

### Principal component analysis

For IRASFS, admixture was estimated using principal components (PCs) from 39 ancestry informative markers (AIMs) and including HapMap CEU and YRI samples for comparison [20]. Only PC1 correlated with HapMap populations, and was thus used as a covariate in all analyses.

For the T2D studies, PCs were computed for each study using high-quality SNPs as previously described [13,17–19,21]. The first PC was highly correlated (r^2^ >0.87) with global African-European ancestry, as measured by ANCESTRYMAP [22], STRUCTURE [23], or FRAPPE [24]. The African American T2D study samples had an average of 80% African ancestry. By analyzing unrelated samples from all studies using SMARTPCA [21], only the first PC appeared to account for substantial genetic variation (data not shown), whereas the subsequent PCs may reflect sampling noise and/or relatedness in samples [22]. The first PC (PC1) was used as a covariate in all analyses to adjust for population substructure.

### Analysis of association with measures of glucose homeostasis in IRASFS

To approximate a normal distribution, trait values were transformed by square root (AIR_g_, DI) or natural logarithm plus a constant (S_I_). Measured genotype association analyses of exome chip variants with AIR_g_, S_I_, and DI were performed under an additive model using the variance components method implemented in Sequential Oligogenic Linkage Analysis Routines (SOLAR) [25] with adjustment for age, gender, body mass index (BMI), and PC1.

### Genetic risk score construction

We further explored our interaction approach by constructing genetic risk scores (GRS), both weighted and unweighted, summarizing the effects of selected AIR_g_ SNPs. The AIR_g_ GRS was created using the AIR_g_-lowering effect alleles for AIR_g_ SNPs (i.e. reflecting poorer insulin secretion) from IRASFS (Table 1). The unweighted risk scores were calculated by summation of the number of risk alleles for each individual across all selected SNPs. The weighted AIR_g_ GRS was calculated as the sum of risk alleles at each locus multiplied by their AIR_g_ effect size from IRASFS. Missing genotypes for a given SNP were imputed as the average number of risk alleles across all samples. The association of each GRS with both AIR_g_ and DI, a combinatorial measure of first-phase insulin secretion and insulin sensitivity, were evaluated in IRASFS using the variance components method implemented in SOLAR [25] adjusted for age, gender, and ancestry proportions.

**Table 1:** Characteristics and single-SNP association results for AIR_g_ SNPs in IRASFS.

AIR_g_ SNP	Chr	Position^a^	Gene	Effect Allele^b^	Other Allele	EAF^c^	Beta	SE^d^	P
rs1582418	5	159882797	*PTTG1*	C	A	0.66	-3.12	0.86	4.00E-04
rs10792837	11	85905715	*EED*	A	G	0.34	-2.92	0.84	4.20E-04
rs10830963	11	92708710	*MTNR1B*	G	C	0.10	-5.80	1.30	1.20E-05
rs10403702	19	40148584	*LGALS16*	T	C	0.80	-3.54	0.95	2.07E-04
rs3827147	20	256727	*C20orf96*	T	A	0.30	-3.09	0.88	3.19E-04

^a^NCBI build 37.

^b^AIR_g_-lowering allele.

^c^Effect allele frequency.

^d^Standard error.

### Analysis of interaction for T2D risk in the T2D studies

Logistic regression with T2D as the outcome was modeled including genetic main and interaction effects as well as AIR_g_ SNP genotype or GRS, age, sex, and principal component covariates for all samples. Additional models included adjustment for BMI, and individuals with missing values were excluded (n = 110).

$$\;Y=\beta_{0}+\beta_{1}S+\beta_{2}G+\beta_{3}S\times G+\beta_{4}C$$ (1)

*Y* is the log odds of T2D, *S* is the genotype for the AIR_g_ SNP or the GRS, *G* is the genotype of the genomic variant, and *C* is the vector of all remaining covariates. The AIR_g_ SNP genotype, both in single-variant analysis and GRS construction, were additively coded with values of 0, 1, and 2 corresponding to dosage of the AIRg-lowering allele. Genomic variant genotypes were additively coded with values of 0, 1, and 2 corresponding to an arbitrarily selected allele dosage of measured genotype or best-guess imputed genotype. Hypothesis testing included a Wald test statistic following a chi-squared distribution with 1 degree-of-freedom under the null *H*_0_:*β*_3_ = 0. In each study, PLINK was used to calculate the interaction effect *β*_3_. Interaction results with extreme values (absolute β or SE>10), primarily due to low cell counts, were excluded. A large number of SNPs with β and SE outliers were excluded in interaction analyses with the AIR_g_ SNP rs1552224 (2,466,070 to 2,479,156 SNPs excluded) due to its low frequency in the T2D study samples (combined MAF = 0.03). For interaction analyses with all other SNPs and risk scores, the number of SNPs excluded as outliers ranged from 0 to 17,000. Interaction results were combined by fixed-effect inverse variance weighting for each candidate SNP or GRS in METAL (http://www.sph.umich.edu/csg/abecasis/metal/). Each meta-analysis contained results for 486,148 to 2,965,304 SNPs.

## Results

### Candidate insulin secretion SNP selection

The characteristics of IRASFS subjects are shown in S1 Table. Samples included 492 African Americans with mean age 41.2 years and mean BMI 29.1 kg/m^2^. Average African ancestry proportion was 0.75. FSIGT was performed for all subjects without T2D (n = 492) to assess measures including insulin secretion (AIR_g_), insulin sensitivity index (S_I_), and disposition index (DI).

Directly genotyped SNPs from the exome chip were tested for association with insulin secretion (AIRg) in IRASFS. Initially, five independent SNPs, which showed evidence of association with AIRg, were selected (effect sizes = -2.92 to -5.80). The MTNR1B SNP, rs10830963 is a well-documented locus associated with fasting glucose and T2D risk in Europeans. It was most powerfully associated with AIRg in IRASFS (*P* = 1.20x10^-5^, Table 1). An additional four variants with the strongest AIRg associations in IRASFS (*P*<5x10^-4^) and MAF≥0.2 (rs1582418, rs10792837, rs10403702, and rs3827147) were also selected (Table 1).

### Interaction analysis

The selected SNPs were examined for genome-wide first order multiplicative interactions with 1) individual insulin secretion SNPs and 2) risk scores summarizing these insulin secretion SNPs. To maximize power, these analyses were performed in five studies (ARIC, CARDIA, JHS, MESA, and WFSM) including 2,725 T2D cases and 4,167 non-diabetic controls and results were meta-analyzed. Representative meta-analysis q-q plots are provided in S1 and S2 Figs. The magnitude of interaction effects cannot be interpreted in terms of disease risk because one must consider the context of marginal effects from each locus when an interaction exists. For this reason, interaction effects have been presented as beta values, not odds ratios, in Tables 2 and 3.

**Table 2:** Top meta-analyzed interactions with AIR_g_ SNPs regressed on T2D risk in ARIC, CARDIA, JHS, MESA, and WFSM.

AIR_g_ SNP (Nearest Gene)	Intxn SNP^a^ (Nearest Gene)	Chr	Position^b^	MAF^c^	β_intxn_^d^	P_intxn_^d^	P_het_^e^	β_intxn_adj_bmi_^f^	P_intxn_adj_bmi_^f^
rs10403702 (*LGALS16*)	rs12026223 (*DPYD*)	1	97866857	0.16	0.53	1.11E-06	0.85	0.53	1.98E-04
rs10403702 (*LGALS16*)	rs10746381 (*LYPLAL1*)	1	219106550	0.46	-0.33	4.43E-06	0.38	-0.32	1.75E-03
rs10403702 (*LGALS16*)	rs17044602 (*DPP10*)	2	116273665	0.10	0.54	3.73E-06	0.51	0.54	2.43E-04
rs10403702 (*LGALS16*)	rs3822387 (*ARHGAP26*)	5	142488305	0.45	0.35	5.05E-07	0.97	0.33	2.16E-04
rs10403702 (*LGALS16*)	rs4975846 (*MRPL36*)	5	1794921	0.32	-0.35	1.63E-06	0.21	-0.36	4.94E-05
rs10403702 (*LGALS16*)	rs2201886 (*NOX3*)	6	155999177	0.16	-0.47	1.40E-06	0.95	-0.49	1.66E-04
rs10403702 (*LGALS16*)	rs1655028 (*SNTB1*)	8	122048009	0.44	0.34	2.21E-06	0.38	0.36	7.34E-06
rs10403702 (*LGALS16*)	rs244783 (*WFDC1*)	16	84360055	0.47	-0.32	3.07E-06	0.96	-0.33	1.11E-04
rs10792837 (*EED*)	rs7796525 (*CHN2*)	7	29410867	0.10	-0.71	1.53E-06	0.27	-0.70	5.39E-04
rs10792837 (*EED*)	rs1342119 (Intergenic)	9	104799619	0.26	0.35	2.91E-06	0.49	0.35	2.12E-03
rs10792837 (*EED*)	rs4556497 (Intergenic)	11	25663415	0.48	-0.56	4.19E-07	0.80	-0.58	9.73E-06
rs10792837 (*EED*)	rs1408201 (*GJA3*)	13	20741094	0.34	0.46	4.14E-06	0.83	0.47	1.53E-04
rs10792837 (*EED*)	rs12978873 (*ZNF761*)	19	53928911	0.24	-0.40	3.36E-06	0.46	-0.39	3.84E-04
rs10830963 (*MTNR1B*)	rs4289500 (*EXOC1*)	4	56638538	0.44	-0.56	4.60E-06	0.58	-0.38	5.19E-02
rs10830963 (*MTNR1B*)	rs2640666 (*MTRR*)	5	7926642	0.38	0.58	3.20E-06	0.66	0.42	2.42E-02
rs10830963 (*MTNR1B*)	rs7277627 (*LCA5L*)	21	40813558	0.34	-0.63	1.65E-06	0.98	-0.44	2.25E-02
rs1582418 (*PTTG1*)	rs2781575 (*ARHGAP29*)	1	94619077	0.07	0.63	4.02E-06	0.83	0.60	5.67E-03
rs1582418 (*PTTG1*)	rs7587317 (*CYS1*)	2	10234179	0.29	-0.31	4.77E-06	0.28	-0.28	2.88E-02
rs1582418 (*PTTG1*)	rs16924460 (*KIAA1217*)	10	24561095	0.08	-0.60	1.70E-07	0.55	-0.65	6.84E-04
rs1582418 (*PTTG1*)	rs6575130 (*CALM1*)	14	90849847	0.45	0.31	1.88E-06	0.23	0.31	8.63E-03
rs1582418 (*PTTG1*)	rs7150527 (*TCL1B*)	14	96125876	0.20	-0.39	3.71E-06	0.33	-0.39	6.71E-03
rs1582418 (*PTTG1*)	rs12597244 (*BC108660*)	16	5776269	0.37	-0.35	8.37E-07	0.65	-0.36	2.47E-03
rs3827147 (*C20orf96*)	rs10975898 (*KDM4C*)	9	6919984	0.22	-0.37	3.72E-06	0.70	-0.35	6.36E-04
rs3827147 (*C20orf96*)	rs10483995 (*KCNK10*)	14	88716607	0.07	0.81	8.75E-07	0.83	0.81	8.47E-05

^a^SNP interacting with selected AIR_g_ SNP with nearest gene within 500 kb in parentheses.

^b^NCBI build 37.

^c^Minor allele frequency.

^d^Meta-analyzed effect size and p-value from interaction models adjusted for age, gender, and PC1.

^e^Heterogeneity p-values across studies from interaction models adjusted for age, gender, and PC1.

^f^Meta-analyzed effect size and p-value from interaction models adjusted for age, gender, PC1, and BMI.

**Table 3:** Top meta-analyzed interactions with weighted AIR_g_ GRS regressed on T2D risk in ARIC, CARDIA, JHS, MESA, and WFSM.

Intxn SNP^a^ (Nearest Gene)	Chr	Position^b^	MAF^c^	β_intxn_^d^	P_intxn_^d^	P_het_^e^	β_intxn_adj_bmi_^f^	P_intxn_adj_bmi_^f^
rs4975846 (*MRPL36*)	5	1794921	0.32	0.05	2.03E-07	0.86	0.05	1.28E-06
rs17074194 (*UTRN*)	6	145107349	0.09	-0.08	1.87E-06	0.71	-0.07	4.02E-05
rs10274367 (*C7orf50*)	7	1117436	0.40	-0.05	1.41E-06	0.47	-0.04	9.63E-06
rs4921630 (Intergenic)	8	16445870	0.06	-0.11	3.98E-06	0.61	-0.12	4.90E-06
rs11625046 (*SRP54*)	14	35497011	0.10	-0.07	4.86E-06	0.68	-0.07	4.32E-05
rs799466 (*SRP54*)	14	35503326	0.10	-0.07	4.53E-06	0.53	-0.07	3.80E-05
rs11627203 (*SAMD4A*)	14	55221543	0.16	-0.07	6.57E-07	0.07	-0.06	1.81E-05

^a^SNP interacting with the weighted AIR_g_ GRS with nearest gene within 500 kb in parentheses.

^b^NCBI build 37.

^c^Minor allele frequency.

^d^Meta-analyzed effect size and p-value from interaction models adjusted for age, gender, and PC1.

^e^Heterogeneity p-values across studies from interaction models adjusted for age, gender, and PC1.

^f^Meta-analyzed effect size and p-value from interaction models adjusted for age, gender, PC1, and BMI.

The characteristics of T2D case (n = 2,725) and control subjects (n = 4,167) for each study cohort are shown in S2 Table. Mean age at examination ranged from 38.2 (CARDIA) to 67.6 (MESA) years. Mean age at diagnosis for T2D cases ranged from 35.0 (CARDIA) to 54.6 (MESA) years. In all cohorts except WFSM, BMI was >3 kg/m^2^ higher in cases compared to controls.

### AIR_g_ SNP interactions

Five AIR_g_ SNPs were tested for genome-wide interactions for T2D risk in the ARIC, CARDIA, JHS, MESA, and WFSM cohorts. Individual AIR_g_ SNP results were meta-analyzed across cohorts. No interactions reached conventional GWAS thresholds for significance (*P*_interaction_<5×10^−8^). However, a total of 24 SNP-pairs were observed with suggestive interaction signals (*P*_interaction_<5×10^−6^; Table 2). The most significant AIR_g_ SNP interaction observed was between rs1582418 at the *PTTG1* locus (AIR_g_ SNP) and rs16924460 (interacting SNP; *P* = 1.70x10^-7^). This interacting SNP is an intronic variant in the gene *KIAA1217*, which encodes the human homolog of murine Skt (Sickle tail). BMI adjustment of the top AIR_g_ SNP interactions resulted in attenuation of the interaction p-value by three to four orders of magnitude for most SNPs (Table 2). Other notable interacting SNPs included rs10746381 (*LYPLAL1*), rs7796525 (*CHN2*), rs4289500 (*EXOC1*), rs10483995 (*KCNK10*), rs6575130 (*CALM1*), rs4975846 (*MRPL36*), rs2640666 (*MTRR*), rs10975898 (*KDM4C*), and rs1655028 (*SNTB1*).

### GRS validation and interaction analysis

Each GRS was tested for association with AIR_g_ and DI under an additive model using the variance components method with adjustment for age, gender, and PC1 in IRASFS (S3 Table). The weighted AIR_g_ GRS was associated with AIR_g_ in IRASFS with or without BMI adjustment (*P* = 4.96x10^-2^ and *P* = 3.34x10^-2^, respectively). Since the weighted risk score was associated with measures of glucose homeostasis, analysis of this risk score was emphasized in the tests for genome-wide interaction in the ARIC, CARDIA, JHS, MESA, and WFSM cohorts.

Meta-analyzed estimates of genome-wide interactions with the weighted AIR_g_ GRS are presented in Table 3. No interactions met conventional GWAS thresholds for significance. However, seven interactions with the weighted AIR_g_ GRS reached a suggestive level of significance (*P*_interaction_ <5x10^-6^; Table 3). In the AIR_g_ GRS interaction analysis the most significant association was observed with interacting SNP rs4975846 (Table 3; *P*_interaction_ = 2.03x10^-7^). This is an intergenic SNP upstream of the gene *MRPL36*, which encodes mitochondrial ribosomal protein L36. This same interacting SNP was implicated in single variant analyses with AIR_g_ SNP rs10403702 (*LGALS16*). Other notable interacting SNPs included rs11627203 (*SAMD4A*, *P*_interaction_ = 6.57x10^-7^) and rs17074194 (*UTRN*, *P*_interaction_ = 1.87x-10^−6^). Top interactions with the AIR_g_ GRS remained robust after BMI adjustment.

For comparison purposes, meta-analysis of single-SNP association performed under an additive logistic regression model with adjustment for age, sex, and principal component covariates was conducted for all interaction SNPs presented in Tables 2 and 3 (S4 Table). None of these SNPs had an association *P*<0.05.

## Discussion

Meta-analyses of five African American T2D studies did not reveal statistically significant first-order interactions with insulin secretion SNPs or composite risk scores. However, the observed interactions (*P*_interaction_<5×10^−6^) suggest that a candidate insulin secretion SNP/GRS interaction approach is a valid method for identifying insulin sensitivity and T2D risk loci. For example, analyses with the AIR_g_ SNP rs10403702 (*LGALS16*) revealed an interaction with rs10746381, an intergenic SNP upstream of the *LYPLAL1* gene encoding lysophospholipase-like 1. Variants at this locus have previously been associated with T2D, fasting insulin, HDL-cholesterol, triglycerides, and measures of body fat distribution, a signature common to insulin sensitivity loci [26–29]. The AIR_g_ SNP rs10792837 (*EED*) showed interaction with rs7796525, an intronic SNP in *CHN2*. Interaction of *CHN2* with the insulin receptor gene *INSR* results in severe insulin resistance [30]. Additionally the AIR_g_ SNP rs10830963 (*MTNR1B*) displayed interaction with rs4289500, an intergenic SNP upstream of *EXOC1*, which encodes a component of the exocyst complex, required for translocation of glucose transporter type 4 (GLUT4) vesicles to the plasma membrane in insulin-stimulated glucose uptake [31].

Several genes related to pancreatic beta-cell function were also identified; suggesting interactions are not limited to insulin resistance as in our initial hypothesis. Analyses with AIR_g_ SNP rs3827147 (*C20orf96*) revealed interactions with rs10483995, an intronic SNP in *KCNK10*. A study of pancreatic beta-cell line MIN6 cells has shown that Kcnk10, a member of the two-pore domain potassium channels, may regulate depolarization-induced secretion of insulin in these cells [32]. The AIR_g_ SNP rs1582418 (*PTTG1*) interaction analyses identified an interaction with an intergenic SNP upstream of *CALM1* (rs6575130). Inhibition of the product of this gene, calmodulin, with phenothiazines inhibits glucose-stimulated insulin secretion in isolated rat islet cells [33–37]. Several other variants detected in our analyses show interactions with similar biological relationships to insulin secretion and T2D.

Interestingly, we observed interactions discrete for individual loci. For example, analyses with rs10830963 (*MTNR1B*) revealed an interaction with rs2640666, an intronic variant downstream of *MTRR*. *MTRR* encodes 5-methyltetrahydrofolate-homocysteine methyltransferase reductase, implicated in metabolic syndrome [38] and epigenetic instability [39]. *MTRR* is highly expressed in the pineal gland in a circadian pattern [40], an intriguing result considering that the ligand for melatonin receptor 1B (encoded by *MTNR1B*), melatonin, is secreted from the pineal gland in a circadian pattern. Also revealed with rs10830963 was an association with rs4289500, which is ~200 kb upstream of *CLOCK*, a classic circadian clock gene. Further, analyses with other insulin secretion SNPs revealed genes involved in epigenetic modification. Analyses with rs3827147 (*C20orf96*) revealed an interaction with rs10975898, an intronic SNP in *KDM4C* which encodes a lysine-specific demethylase. We also identified 2 dystrophin-related genes. Analyses with rs10403702 (*LGALS16*) revealed an interaction with rs1655028, an intronic SNP upstream of *SNTB1*. This gene encodes beta 1 syntrophin, a peripheral membrane protein that associates with dystrophin and dystrophin-related proteins. Evaluations of the AIR_g_ GRS revealed an interaction with rs17074194, an intronic SNP in *UTRN*. This gene encodes utrophin, a cytoskeletal protein which has homology with dystrophin and is found at the neuromuscular synapse and myotendinous junctions in muscle cells. These observations may reflect different, input-dependent physiological characteristics of interaction results, and may lead to mechanistic insights about the underlying causes of T2D and defects in glucose homeostasis in expanded analyses.

Although results varied widely between interaction analyses, interaction with one locus, *MRPL36*, was replicated in multiple analyses. Functional characteristics of *MRPL36* related to T2D and glucose homeostasis pathophysiology are not evident in the current literature.

Previous GWAS have largely ignored epistatic contributions to T2D risk due to the heavy multiple testing burden and computational challenges of exhaustive analytical approaches, and when they have considered this contribution, results have not been striking. For example, a recent genome-wide scan for two-locus interactions in the Wellcome Trust Case Control Consortium T2D GWAS data did not reveal any significant epistatic signals at a Bonferroni-corrected p-value threshold of 2.14x10^-11^ after adjusting for the main effects of the most strongly associated T2D locus, *TCF7L2* [41]. Further, Herold et al. estimated that analysis of all pairwise interactions among 550,000 SNPs in 1,200 samples on a 3 GHz computer would require a running time of 120 days [42]. The interaction analysis presented here overcomes the issue of a heavy multiple testing burden by using a candidate SNP approach. A recent study by Becker et al. demonstrated that a multiple test correction of 0.4m, where m is the number of SNP pairs tested, is sufficiently conservative for large-scale allelic interaction tests [43]. Further, Babron et al. show that a correction for the effective number of SNP pairs is equally sufficient [44]. Li et al. previously demonstrated that the effective number of SNPs for an imputed dataset is ~10^6^. These findings suggest that a significance threshold of 1x10^-8^ is appropriate for this study.

We did not detect interactions even at the conventional GWAS threshold of 5x10^-8^ in the current study. In part, this likely reflects the challenge of inherently reduced power of interaction models due to the low frequency of compound genotypes [45]. Computational resources required for this study were equivalent to the requirements for running 12 GWAS (5 candidate insulin secretion SNPs plus a GRS, with and without BMI adjustment). This is a significant reduction compared to exhaustive approaches examining genome-wide interactions with all available SNP pairs. Although the results of a meta-analysis of five cohorts are presented here, this study lacks replication of the observed interaction associations in additional studies. Future work will require examination of associated interactions in African American and other populations.

In summary, our findings demonstrate that genome-wide interaction studies with selected insulin secretion variants is a powerful approach for the detection of T2D risk, insulin secretion, and insulin sensitivity loci. The use of a high-quality measure of first-phase insulin secretion, AIR_g_, to identify candidate interaction SNPs yielded compelling associations. These results justify an expansion of the current study and further investigation of putative insulin sensitivity loci, namely *LYPLAL1*, *CHN2*, and *EXOC1*.

## Supporting Information

S1 Appendix Descriptions of the T2D study cohorts. (DOCX)

S1 Table Descriptive characteristics of IRASFS African Americans. *Data are shown as count, mean, percentage, or mean ± SD or percentage.
(DOCX)

S2 Table Descriptive characteristics of African American diabetes case and control subjects. Data are shown as count, percentage, or mean ± SD. *Age and BMI are shown for the last available visit for the prospective studies including ARIC, CARDIA, and MESA (Exam 4); and the baseline visit for JHS and WFSM.
(DOCX)

S3 Table Association of AIR_g_ GRS with AIR_g_ and DI in IRASFS. *Model 1 is adjusted for age, gender, and PC1. †Model 2 is adjusted for age, gender, PC1, and BMI.
(DOCX)

S4 Table Single-SNP association results for interacting SNPs. ^a^SNP interacting with the selected AIR_g_ SNP or the weighted AIR_g_ GRS with nearest gene within 500 kb in parentheses. ^b^Meta-analyzed effect size, standard error, and p-value from association models adjusted for age, gender, and PC1. ^c^Heterogeneity p-values across studies from association models adjusted for age, gender, and PC1.
(DOCX)

S1 Fig Q-Q plot for meta-analyzed interactions with AIR_g_ SNP rs10830963 (*MTNR1B*) regressed on T2D risk in ARIC, CARDIA, JHS, MESA, and WFSM from interaction models adjusted for age, gender, and PC1. (TIF)

S2 Fig Q-Q plot for meta-analyzed interactions with weighted AIR_g_ GRS regressed on T2D risk in ARIC, CARDIA, JHS, MESA, and WFSM from interaction models adjusted for age, gender, and PC1. (TIF)
