# Supplementary material for: Genome-Wide Interaction with Insulin Secretion Loci Reveals Novel Loci for Type 2 Diabetes in African Americans
Source: PLoS One. 2016 Jul 22;11(7):e0159977. doi: 10.1371/journal.pone.0159977 (PMC4957757; doi:10.1371/journal.pone.0159977)
Supplement: S1 Appendix — (DOCX) [file pone.0159977.s001.docx]

**S1 Appendix**

As described in detail previously[1], the following is a summary of funding sources and study characteristics for each of the T2D study cohorts.

**Funding**

Genotyping services for the WFSM study were provided by CIDR. CIDR is fully funded through a federal contract from the National Institutes of Health (NIH) to The Johns Hopkins University (Contract HHSC268200782096C). The work at Wake Forest was supported by NIH grants K99-DK-081350 (N.D.P.), R01-DK-066358 (D.W.B.), R01-DK-053591 (D.W.B.), R01-HL-56266 (B.I.F.), and R01-DK-070941 (B.I.F.), and in part by the General Clinical Research Center of the WFSM Grant M01-RR-07122. This work was also supported by the NHLBI.

The following four parent studies have contributed parent study data, ancillary study data, and DNA samples through the Massachusetts Institute of Technology-Broad Institute (N01-HC-65226) to create this genotype/phenotype database for wide dissemination to the biomedical research community:

ARIC, CARDIA, JHS, and MESA.

The Atherosclerosis Risk in Communities (ARIC) Study is carried out as a collaborative study supported by National Heart, Lung, and Blood Institute contracts (HHSN268201100005C, HHSN268201100006C, HHSN268201100007C, HHSN268201100008C, HHSN268201100009C, HHSN268201100010C, HHSN268201100011C, and HHSN268201100012C), R01HL087641, R01HL59367 and R01HL086694; National Human Genome Research Institute contract U01HG004402; and National Institutes of Health contract HHSN268200625226C. The authors thank the staff and participants of the ARIC study for their important contributions. Infrastructure was partly supported by Grant Number UL1RR025005, a component of the National Institutes of Health and NIH Roadmap for Medical Research. The authors thank the staff and participants of the ARIC study for their important contributions.

The Coronary Artery Risk Development in Young Adults (CARDIA) Study is conducted and supported by the National Heart, Lung, and Blood Institute in collaboration with the University of Alabama at Birmingham (HHSN268201300025C & HHSN268201300026C), Northwestern University (HHSN268201300027C), University of Minnesota (HHSN268201300028C), Kaiser Foundation Research Institute (HHSN268201300029C), and Johns Hopkins University School of Medicine (HHSN268200900041C). CARDIA is also partially supported by the Intramural Research Program of the National Institute on Aging. Genotyping was funded as part of the NHLBI Candidate-gene Association Resource (N01-HC-65226) and the NHGRI Gene Environment Association Studies (GENEVA) (U01-HG004729, U01-HG04424, and U01-HG004446). This manuscript has been reviewed and approved by CARDIA for scientific content.

The Jackson Heart Study (JHS) is supported by contracts HHSN268201300046C, HHSN268201300047C, HHSN268201300048C, HHSN268201300049C, HHSN268201300050C from the National Heart, Lung, and Blood Institute and the National Institute on Minority Health and Health Disparities. The authors thank the participants and data collection staff of the Jackson Heart Study.

Multi-Ethnic Study of Atherosclerosis (MESA), and the MESA SHARe project are conducted and supported by the National Heart, Lung, and Blood Institute (NHLBI) in collaboration with MESA investigators. Support for MESA is provided by contracts N01-HC-95159, N01-HC-95160, N01-HC-95161, N01-HC-95162, N01-HC-95163, N01-HC-95164, N01-HC-95165, N01-HC-95166, N01-HC-95167, N01-HC-95168, N01-HC-95169, UL1-TR-001079, UL1-TR-000040, and DK063491. The MESA CARe data used for the analyses described in this manuscript were obtained through Genetics (CMP00068). Funding for CARe genotyping was provided by NHLBI Contract N01-HC-65226.

The views expressed in this manuscript are those of the authors and do not necessarily represent the views of the National Heart, Lung, and Blood Institute; the National Institutes of Health; or the U.S. Department of Health and Human Services.

**Descriptions of the T2D study cohorts**

**ARIC**

The ARIC study is a prospective population-based study of atherosclerosis and cardiovascular disease that included 15792 participants (27% African American) aged 45-64 years at baseline visit (1987-89, visit 1) from four US communities [2]. Participants completed follow-up visits in 1990-92 (visit 2), 1993-95 (visit 3), 1996-98 (visit 4), and 2011-13 (visit 5). In this study, 955 T2D subjects diagnosed at any of the first four visits (visits 1-4) and 414 subjects with normal glucose tolerance (NGT) at visits 1-4 were included. All subjects were self-reported African American recruited from two communities (Jackson, MS and Forsyth, NC).

**CARDIA**

The CARDIA study is a prospective multi-center investigation of the natural history and etiology of cardiovascular disease that included 5115 participants (52% African American) aged 18-30 years at baseline visit from four US communities (Birmingham, AL; Chicago, IL; Minneapolis, MN and Oakland, CA) [3]. Follow-up examinations occurred at years 2, 5, 7, 10, 15 and 20. In this study, 94 T2D subjects diagnosed at any visits and 654 subjects with NGT in all visits were included. All subjects were self-reported African American.

**JHS**

JHS is a prospective population-based study to examine the risk factors of cardiovascular diseases among 5301 African American from two cohorts of unrelated (aged 35-84 years) and nested family-based (aged ≥21 years) subjects in the Jackson, Mississippi metropolitan area [4]. The mean family size was 1.4±1.5 subjects per family. In this study, 333 T2D and 1450 NGT subjects at baseline visit who were not enrolled in the ARIC study were included. The respective mean family size was 1.3±1.4 subjects per family, and 88% of families are singletons. Family relationship was not accounted during association analysis due to the low degree of relatedness.

**MESA**

MESA is a prospective community-based study of the characteristics of subclinical cardiovascular disease and included 6,814 individuals (28% African American) free from known cardiovascular disease between 45–84 years old at baseline [5]. Subjects were recruited from six field centers (Wake Forest School of Medicine, Columbia University, Johns Hopkins University, University of Minnesota, Northwestern University and University of California - Los Angeles). Data from up to the fifth visit are available for analyses (Exam 1 2000-02, Exam 2 2002-04, Exam 3 2004-05, Exam 4 2005-07, Exam 5 2010-12). In this study, 411 T2D subjects diagnosed in any visit up to Exam 4 and 793 subjects with NGT in all visits up to Exam 4 who were self-reported African American were included.

**WFSM**

The WFSM study is a cross-sectional case-control study designed to examine the genetics of T2D and end-stage renal disease (ESRD) in African American [6,7]. In this study, the cases included 932 subjects with both T2D and ESRD recruited from dialysis facilities. In addition, cases had at least one of the following inclusion criteria: a) T2D diagnosed at least 5 years before initiating renal replacement therapy, b) background or greater diabetic retinopathy and/or c) ≥100 mg/dl proteinuria on urinalysis in the absence of other causes of nephropathy. The controls included 856 African American subjects without a current diagnosis of diabetes or renal disease recruited from the community and internal medicine clinics. All subjects were recruited in North Carolina, South Carolina, Georgia, Tennessee or Virginia.

**References**

1. Ng MCY, Saxena R, Li J, Palmer ND, Dimitrov L, Xu J, et al. Transferability and fine mapping of type 2 diabetes loci in African Americans: the Candidate Gene Association Resource Plus Study. Diabetes. 2013;62: 965–976. doi:10.2337/db12-0266

2. The ARIC Investigators. The Atherosclerosis Risk in Communities (ARIC) Study: design and objectives. The ARIC investigators. Am J Epidemiol. 1989;129: 687–702.

3. Friedman GD, Cutter GR, Donahue RP, Hughes GH, Hulley SB, Jacobs DR, et al. CARDIA: study design, recruitment, and some characteristics of the examined subjects. J Clin Epidemiol. 1988;41: 1105–1116.

4. Taylor HA, Wilson JG, Jones DW, Sarpong DF, Srinivasan A, Garrison RJ, et al. Toward resolution of cardiovascular health disparities in African Americans: design and methods of the Jackson Heart Study. Ethn Dis. 2005;15: S6-4–17.

5. Bild DE, Bluemke DA, Burke GL, Detrano R, Diez Roux AV, Folsom AR, et al. Multi-ethnic study of atherosclerosis: objectives and design. Am J Epidemiol. 2002;156: 871–881.

6. McDonough CW, Palmer ND, Hicks PJ, Roh BH, An SS, Cooke JN, et al. A genome-wide association study for diabetic nephropathy genes in African Americans. Kidney Int. 2011;79: 563–572. doi:10.1038/ki.2010.467

7. Palmer ND, McDonough CW, Hicks PJ, Roh BH, Wing MR, An SS, et al. A genome-wide association search for type 2 diabetes genes in African Americans. PloS One. 2012;7: e29202. doi:10.1371/journal.pone.0029202
