# Supplementary material for: Genome-Wide Interaction with Insulin Secretion Loci Reveals Novel Loci for Type 2 Diabetes in African Americans
Source: PLoS One. 2016 Jul 22;11(7):e0159977. doi: 10.1371/journal.pone.0159977 (PMC4957757; doi:10.1371/journal.pone.0159977)
Supplement: S1 Table — *Data are shown as count, mean, percentage, or mean ± SD or percentage. (DOCX) [file pone.0159977.s002.docx]

**S1 Table.** Descriptive characteristics of IRASFS African Americans.

| **Characteristic*** | **Value** |
| --- | --- |
| N | 492 |
| Pedigrees | 42 |
| Mean pedigree size | 11.7 |
| Male (%) | 42.4 |
| Age (years) | 41.2±13.7 |
| Acute Insulin Response (µU ml^-1^ min) | 1002.2±820.9 |
| Insulin Sensitivity (x10^-4^ min^-1^ µU^-1^ ml) | 1.6±1.2 |
| Disposition Index | 1425.5±1273.7 |
| BMI (kg/m^2^) | 29.1±5.1 |
| African ancestry proportion | 0.75±0.12 |

*Data are shown as count, mean, percentage, or mean ± SD or percentage
