# Supplementary material for: Genome-Wide Interaction with Insulin Secretion Loci Reveals Novel Loci for Type 2 Diabetes in African Americans
Source: PLoS One. 2016 Jul 22;11(7):e0159977. doi: 10.1371/journal.pone.0159977 (PMC4957757; doi:10.1371/journal.pone.0159977)
Supplement: S2 Table — Data are shown as count, percentage, or mean ± SD. *Age and BMI are shown for the last available visit for the prospective studies including ARIC, CARDIA, and MESA (Exam 4); and the baseline visit for JHS and WFSM. (DOCX) [file pone.0159977.s003.docx]

**S2 Table.** Descriptive characteristics of African American diabetes case and control subjects.

| **Characteristic** | **ARIC** | | **CARDIA** | | **JHS** | | **MESA** | | **WFSM** | |
| --- | --- | --- | --- | --- | --- | --- | --- | --- | --- | --- |
|  | **case** | **control** | **case** | **control** | **case** | **control** | **case** | **control** | **case** | **control** |
| N | 955 | 414 | 94 | 654 | 333 | 1450 | 411 | 793 | 932 | 856 |
| Male (%) | 35.8 | 31.2 | 19.2 | 38.2 | 33.9 | 38.7 | 47.2 | 42.4 | 38.8 | 43.7 |
| Age (years) * | 61.3±6.0 | 59.6±6.3 | 40.5±3.8 | 38.2±4.4 | 55.5±10.7 | 48.6±11.3 | 67.6±9.2 | 65.3±10.5 | 61.6 ±10.5 | 49.0±11.9 |
| Age at diagnosis of T2D (years) | 50.9±9.2 | - | 35.0±5.5 | - | 46.2±11.0 | - | 54.6±10.9 | - | 41.6±12.3 | - |
| BMI (kg/m2) * | 32.0±6.7 | 27.6±5.7 | 33.8±8.1 | 29.6±6.8 | 35.2±7.5 | 31.4±7.5 | 31.7±6.2 | 28.6±5.8 | 29.7±7.1 | 30.0±7.1 |
| African ancestry proportion | 0.83±0.10 | 0.83±0.09 | 0.83±0.08 | 0.80±0.11 | 0.83±0.08 | 0.82±0.09 | 0.79±0.14 | 0.78±0.14 | 0.80±0.11 | 0.78±0.11 |

Data are shown as count, percentage, or mean ± SD. *Age and BMI are shown for the last available visit for the prospective studies including ARIC, CARDIA, and MESA (Exam 4); and the baseline visit for JHS and WFSM.
