# Supplementary material for: Genome-Wide Interaction with Insulin Secretion Loci Reveals Novel Loci for Type 2 Diabetes in African Americans
Source: PLoS One. 2016 Jul 22;11(7):e0159977. doi: 10.1371/journal.pone.0159977 (PMC4957757; doi:10.1371/journal.pone.0159977)
Supplement: S3 Table — *Model 1 is adjusted for age, gender, and PC1. †Model 2 is adjusted for age, gender, PC1, and BMI. (DOCX) [file pone.0159977.s004.docx]

**S3 Table.** Association of AIR_g_ GRS with AIR_g_ and DI in IRASFS.

|  | **AIR_g_** | | **DI** | |
| --- | --- | --- | --- | --- |
|  | **Beta** | **P-value** | **Beta** | **P-value** |
| Unweighted |  |  |  |  |
| Model 1* | -0.59 | 1.74E-01 | -0.84 | 1.50E-01 |
| Model 2† | -0.64 | 1.36E-01 | -0.62 | 2.74E-01 |
| Weighted |  |  |  |  |
| Model 1* | -0.23 | 4.96E-02 | -0.31 | 5.11E-02 |
| Model 2† | -0.25 | 3.34E-02 | -0.24 | 1.27E-01 |

*Model 1 is adjusted for age, gender, and PC1. †Model 2 is adjusted for age, gender, PC1, and BMI
