# Supplementary material for: Genome-Wide Interaction with Insulin Secretion Loci Reveals Novel Loci for Type 2 Diabetes in African Americans
Source: PLoS One. 2016 Jul 22;11(7):e0159977. doi: 10.1371/journal.pone.0159977 (PMC4957757; doi:10.1371/journal.pone.0159977)
Supplement: S4 Table — aSNP interacting with the selected AIRg SNP or the weighted AIRg GRS with nearest gene within 500 kb in parentheses. bMeta-analyzed effect size, standard error, and p-value from association models adjusted for age, gender, and PC1. cHeterogeneity p-values across studies from association models adjusted for age, gender, and PC1. (DOCX) [file pone.0159977.s005.docx]

**S4 Table.** Single-SNP association results for interacting SNPs.

| **Intxn SNP^a^ (Nearest Gene)** | **Allele 1** | **Allele 2** | **Allele 1 Freq** | **β_assoc_^b^** | **SE_assoc_^b^** | **P_assoc_^b^** | **P_het_^c^** |
| --- | --- | --- | --- | --- | --- | --- | --- |
| rs12026223 (*DPYD*) | t | c | 0.16 | 0.010 | 0.060 | 0.87 | 0.71 |
| rs10746381 (*LYPLAL1*) | a | g | 0.46 | 0.003 | 0.043 | 0.94 | 0.13 |
| rs17044602 (*DPP10*) | a | g | 0.10 | 0.023 | 0.069 | 0.74 | 0.32 |
| rs3822387 (*ARHGAP26*) | a | g | 0.55 | 0.007 | 0.043 | 0.87 | 0.35 |
| rs4975846 (*MRPL36*) | t | c | 0.68 | -0.088 | 0.044 | 0.05 | 0.65 |
| rs2201886 (*NOX3*) | a | g | 0.16 | 0.012 | 0.057 | 0.83 | 0.80 |
| rs1655028 (*SNTB1*) | t | c | 0.44 | -0.046 | 0.043 | 0.29 | 0.36 |
| rs244783 (*WFDC1*) | t | g | 0.47 | 0.032 | 0.041 | 0.44 | 0.74 |
| rs7796525 (*CHN2*) | a | g | 0.10 | -0.031 | 0.087 | 0.73 | 0.31 |
| rs1342119 (Intergenic) | a | g | 0.26 | 0.008 | 0.048 | 0.87 | 0.83 |
| rs4556497 (Intergenic) | a | g | 0.52 | 0.030 | 0.052 | 0.56 | 0.48 |
| rs1408201 (*GJA3*) | a | t | 0.34 | 0.003 | 0.052 | 0.96 | 0.77 |
| rs12978873 (*ZNF761*) | c | g | 0.24 | -0.030 | 0.051 | 0.55 | 0.47 |
| rs4289500 (*EXOC1*) | c | g | 0.44 | -0.053 | 0.042 | 0.21 | 0.99 |
| rs2640666 (*MTRR*) | t | c | 0.62 | -0.020 | 0.043 | 0.64 | 0.90 |
| rs7277627 (*LCA5L*) | t | g | 0.34 | -0.056 | 0.044 | 0.21 | 0.76 |
| rs2781575 (*ARHGAP29*) | c | g | 0.07 | 0.168 | 0.088 | 0.05 | 0.36 |
| rs7587317 (*CYS1*) | t | c | 0.29 | 0.073 | 0.046 | 0.11 | 0.01 |
| rs16924460 (*KIAA1217*) | a | g | 0.92 | -0.068 | 0.075 | 0.36 | 0.53 |
| rs6575130 (*CALM1*) | a | c | 0.55 | 0.019 | 0.043 | 0.66 | 0.27 |
| rs7150527 (*TCL1B*) | t | c | 0.20 | 0.040 | 0.054 | 0.46 | 0.66 |
| rs12597244 (*BC108660*) | t | c | 0.37 | 0.022 | 0.045 | 0.62 | 0.26 |
| rs10975898 (*KDM4C*) | a | g | 0.78 | -0.034 | 0.049 | 0.49 | 0.10 |
| rs10483995 (*KCNK10*) | a | c | 0.93 | 0.122 | 0.093 | 0.19 | 0.02 |
| rs17074194 (*UTRN*) | t | c | 0.09 | 0.028 | 0.072 | 0.69 | 0.20 |
| rs10274367 (*C7orf50*) | t | c | 0.59 | 0.008 | 0.043 | 0.86 | 0.78 |
| rs4921630 (Intergenic) | t | c | 0.94 | 0.017 | 0.095 | 0.86 | 0.37 |
| rs11625046 (*SRP54*) | t | g | 0.90 | -0.095 | 0.070 | 0.17 | 0.89 |
| rs799466 (*SRP54*) | t | c | 0.90 | -0.105 | 0.070 | 0.14 | 0.84 |
| rs11627203 (*SAMD4A*) | t | c | 0.84 | -0.011 | 0.062 | 0.85 | 0.51 |

^a^SNP interacting with the selected AIR_g_ SNP or the weighted AIR_g_ GRS with nearest gene within 500 kb in parentheses. ^b^Meta-analyzed effect size, standard error, and p-value from association models adjusted for age, gender, and PC1. ^c^Heterogeneity p-values across studies from association models adjusted for age, gender, and PC1.
