# Supplementary figures and images for: Genome-Wide Interaction with Insulin Secretion Loci Reveals Novel Loci for Type 2 Diabetes in African Americans
Source: PLoS One. 2016 Jul 22;11(7):e0159977. doi: 10.1371/journal.pone.0159977 (PMC4957757; doi:10.1371/journal.pone.0159977)

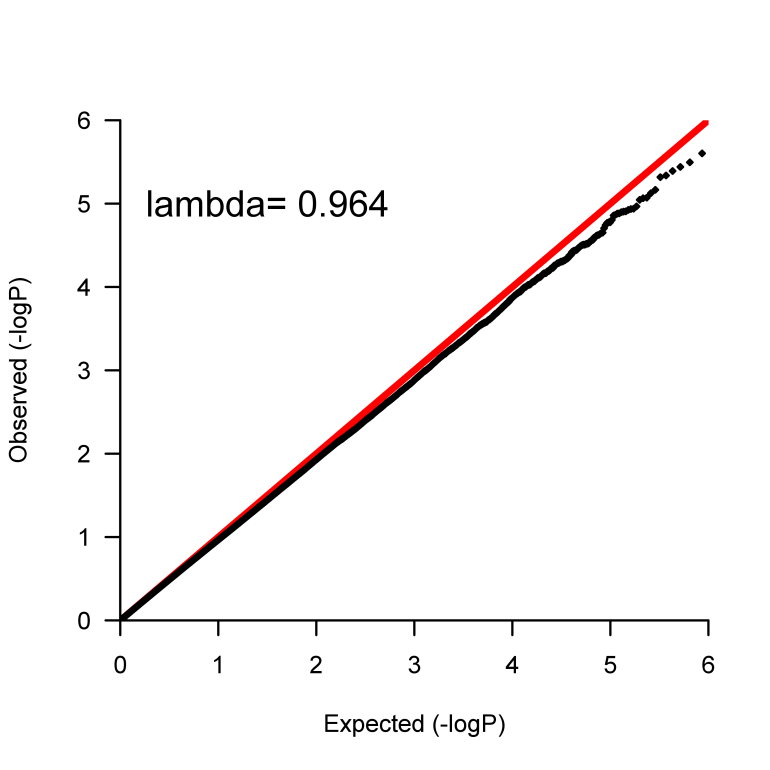

Supplement: S1 Fig — (TIF) [file pone.0159977.s006.tif]

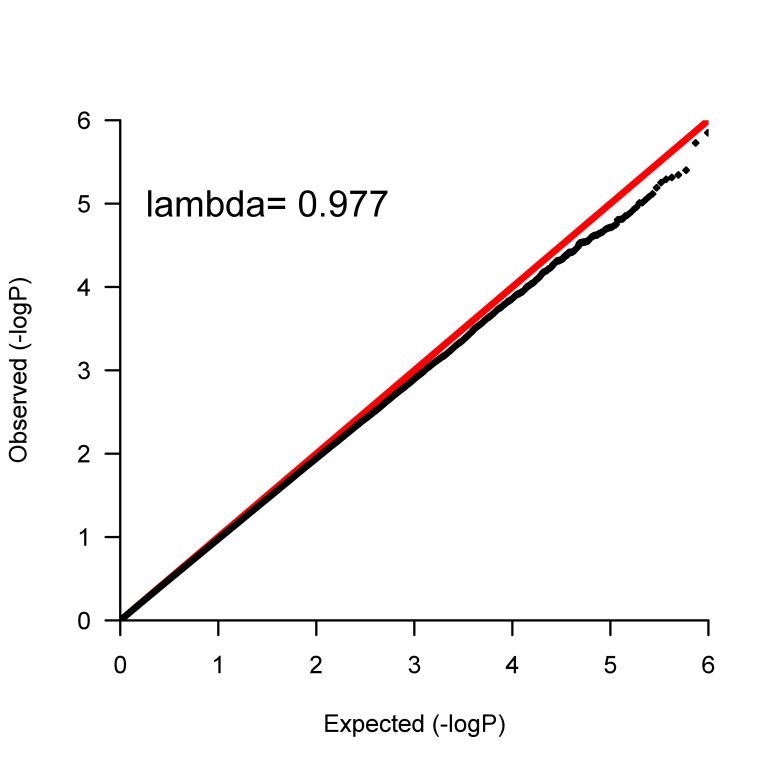

Supplement: S2 Fig — (TIF) [file pone.0159977.s007.tif]
